# How are location and type of caring associated with the carer’s mental health? Cross-sectional and longitudinal findings from SHARE

**DOI:** 10.1007/s10433-025-00843-3

**Published:** 2025-02-21

**Authors:** Valerie Schaps, Thomas Hansen, Ragnhild Bang Nes, Morten Wahrendorf

**Affiliations:** 1 Institute of Medical Sociology, Medical Faculty and University Hospital Düsseldorf, Heinrich Heine University Düsseldorf, Moorenstrasse 5, 40225 Düsseldorf, Germany; 2 Department of Mental Health, Norwegian Institute of Public Health, Oslo, Norway; 3 Promenta Research Center, University of Oslo, Oslo, Norway

**Keywords:** Caring, Mental health, Depressive symptoms, Location of care, Type of care

## Abstract

Research findings on the relationship between caring and health are mixed and call for a more nuanced analysis of the care situation. This study investigates cross-sectional and longitudinal associations between caring and depressive symptoms, considering location and type of care. Data come from the Survey of Health Ageing and Retirement in Europe (SHARE), collected in wave 6 (from 2015 to 2016) and wave 8 (from 2019 to 2020). Cross-sectional data were available for 52.186 respondents in 18 countries. Of these, 18.659 were free of elevated depressive symptoms in wave 6 and were used to investigate incident depression in the longitudinal analyses. We distinguished between personal care, practical help, and paperwork, and were able to compare in-home and outside-home personal care. For both men and women, findings indicate that in-home personal care is associated with an increased risk of reporting and developing depressive symptoms (after controlling for age, country affiliation, education, wealth, employment situation, and functional limitations). Cross-sectional, but not longitudinal, associations were also found for outside-home care in terms of personal care or paperwork, but not for practical help. Additionally, women and disadvantaged population groups were more likely to provide in-home care, but less likely to provide outside care (regardless of the type). Overall, the results highlight that different locations and types of care are associated differently with mental health. Findings also underscore the need for interventions specifically tailored to support disadvantaged populations who provide in-home care, addressing the unique challenges they face.

**Supplementary Information:**

The online version contains supplementary material available at 10.1007/s10433-025-00843-3.

## Introduction

Caring outside a formal setting can be defined as the unpaid assistance provided to people who have difficulties in daily life due to physical, cognitive, or emotional impairments, and includes tasks such as instrumental support (e.g. household help or transport), administrative assistance, and personal care (Hoffmann and Rodrigues 2010; Triantafillou et al. 2010; Eurofamcare 2006). With almost 80% of all care being provided outside a formal setting in Europe, it is an indispensable part of current care provision and an important pillar in meeting the growing demand for care in Europe (Hoffmann and Rodrigues 2010; European Commission 2021). Due to ageing societies, the importance of carers is expected to increase in future (Pavolini and Ranci 2008; Ophir and Polos 2022). Against this background, it is of great importance to take an in-depth look at the prevalence of caring in Europe as well as to study the association between caring and health from the carer’s perspective.

Providing care for a disabled family member is physically and emotionally taxing and may impede engagement in personal, familial, and work-related activities. In addition, it may induce worry concerning one's own capacity to handle future care demands and concern for the health and well-being of the care recipient. However, despite the importance of caring, knowledge about sociodemographic and socioeconomic variations in care provision remains unclear, and the evidence on health-related consequences of caring is mixed. One possible reason for the limited knowledge is the different measures of caring used in existing studies. For example, some studies use broad, undifferentiated measures and fail to distinguish between different types of care (e.g. practical support and personal care). Conversely, studies that focus on a particular type of care allow for examination of variations between carers and non-carers and the health impact of one type only, but fail to provide a systematic comparison of different types of care. Similarly, while some studies consider the location of care and distinguish between care provided inside and outside the household (e.g. De Koker 2009; Hansen et al. 2013), others do not (e.g. De Klerk et al. 2021), even if information on the location is available (e.g. Bom et al. 2019; Stöckel and Bom 2022). In fact, the type and the location of caring may be important aspects of the care situation that are relevant for health-related consequences of the carer. Studies on paid employment, for example, show that the location of work, e.g. home office and a separation between work and home, has a dualistic nature. On the one hand, several authors report positive experiences in association with remote working, while others have not found lasting positive results (for a systematic review see Figueiredo et al. 2024). Likewise, providing personal care within a household setting may involve more intensive caring responsibilities, leading to greater personal restrictions, increased worries, and more profound mental health effects than providing practical assistance outside the household, which is more likely to be provided on a more voluntary and flexible basis. It is the overarching objective of the present paper to shed light on these issues by studying both cross-sectional and longitudinal associations of caring and mental health, considering type and location of caring, among men and women across Europe.

The previous studies on sociodemographic differences in caring suggest that women are more likely to provide care than men, especially in societies with traditional values of women as natural carers (Tur-Sinai et al. 2020; Eurocarers 2021). Furthermore, many studies show that older age is associated with an increasing prevalence of caring, mainly because older people are more likely to have a partner who needs care (Mentzakis et al. 2011). However, there is also some evidence of a decreasing prevalence of caring after the age of 60, especially when using a broader measure of caring that does not focus solely on personal care (Dahlberg et al. 2007). In addition, when looking at socioeconomic differences, there is evidence that people with lower income and lower formal education are more likely to provide care within the household (Quashie et al. 2022; Mentzakis et al. 2011, 2009; De Klerk et al. 2021). But again, other studies with broader measures of care also report a higher prevalence of caring among people with higher education (Tur-Sinai et al. 2020). Overall, this suggests sociodemographic and socioeconomic differences, but also that differentiating between types of care may provide a clearer picture of these differences. Also, beside the aforementioned sex differences, studies suggest that men experience caring (and its potential burden) differently than women (Swinkels et al. 2019; Gallicchio et al. 2002; Zygouri et al. 2021). In sum, these differences suggest that the associations between caring and mental health should be investigated separately for men and women, and that sociodemographic and socioeconomic factors should be taken into account.

The number of studies investigating links between caring and mental health has clearly grown in recent years. Hereby, the evidence on the health-related consequences of caring is rather complex and suggests that the association depends on several aspects of the care situation. Besides the frequency (e.g. Morrow-Howell et al. 2003; Van Willigen 2000; Wahrendorf et al. 2008; Stöckel and Bom 2022), the relationship to the care recipient (of “emotional closeness”) (Litwin and Stoeckel 2014) and the care recipient’s health (Papastavrou et al. 2007), two aspects of key importance are the focus of the present paper: the *location* and the *type* of caring. In the case of the latter, studies suggest that care activities involving personal care tend to be associated with poorer mental health. For example, based on cross-sectional data from the European Social Survey, Verbakel and colleagues reported higher levels of depressive symptoms among people who provided personal care, particular among women and those who provided more than 10 h of care per week (Verbakel et al. 2017). Similarly, a longitudinal study based on the Survey of Health Ageing in Retirement focused on personal care and found that it was associated with poorer both mental and physical carer health, but without examining and comparing the effect for different types of care and without conducting sex-specific analyses (Hiel et al. 2015). The negative association between care provision and carer health is usually explained by increased physical or psychosocial strain (Broese van Groenou et al. 2013; Morasso et al. 2008; Schulz et al. 1997; Biliunaite et al. 2022; Doebler et al. 2017; Verbakel et al. 2017; Roth et al. 2009), especially in terms of restricted control or autonomy (Wahrendorf et al. 2008; Haidt and Rodin 1999), a double burden through care and employment (Bom and Stöckel 2021), or limited reward and recognition (McMunn et al. 2009; Siegrist and Wahrendorf 2009). In terms of the location of care, a number of studies suggest that the location of care also matters. In particular, care within the household tends to be associated with poorer mental health compared to not caring, whereas the opposite is true for care outside the household (Kaschowitz and Brandt 2017; Hansen et al. 2013). This finding is often explained by the fact that care within the household usually involves care for a sick and disabled relative, while the better health of carers outside the household can be explained by their better general health and related selection processes, as well as by the fact that the care situation may involve different, less stressful tasks and even enriching experiences (Hansen et al. 2013). In fact, a comprehensive study of the association between caring and mental health that explicitly considers the type and location of the care situation and takes sex differences into account is still lacking.

Taken together, it is rather surprising that many studies do not consider different caring situations and assess important aspects of the care activity in relation to mental health. Such knowledge would not only deepen our understanding on the association between caring and health, but would also help to identify groups of carers at particular risk of poor health and deserving special attention in intervention efforts. Based on cross-sectional and longitudinal data of the Survey of Health Ageing and Retirement in Europe, the main aim of this paper is to address these important shortcomings.

## Methods

### Data source

We use data from the Survey of Health, Ageing, and Retirement in Europe (SHARE, Release 8.0.0). SHARE is the first cross-national, longitudinal research project collecting data on a variety of sociological, economic, and health-related topics at 2-year intervals among older people across Europe. In each country, the sample is based on probability household samples where people above 50 years were interviewed plus their (possibly younger) partners in the household using Computer-Assisted Personal Interviews (CAPI). First wave data were collected 2004 in eleven European countries (plus Israel). Additional countries have joined SHARE over time, and most countries include new participants in the course of the survey, allowing to increase the sample size and to maintain population representation (based on so-called “refreshment samples”). Latest data are available from wave 9 in 28 European countries (plus Israel). Wave 3 and wave 7 of SHARE consist of separate retrospective assessments of respondents' previous life, with limited information on current information. More details about SHARE are available elsewhere (Börsch-Supan et al. 2013; SHARE 2022).

### Study population

**Fig. 1:**
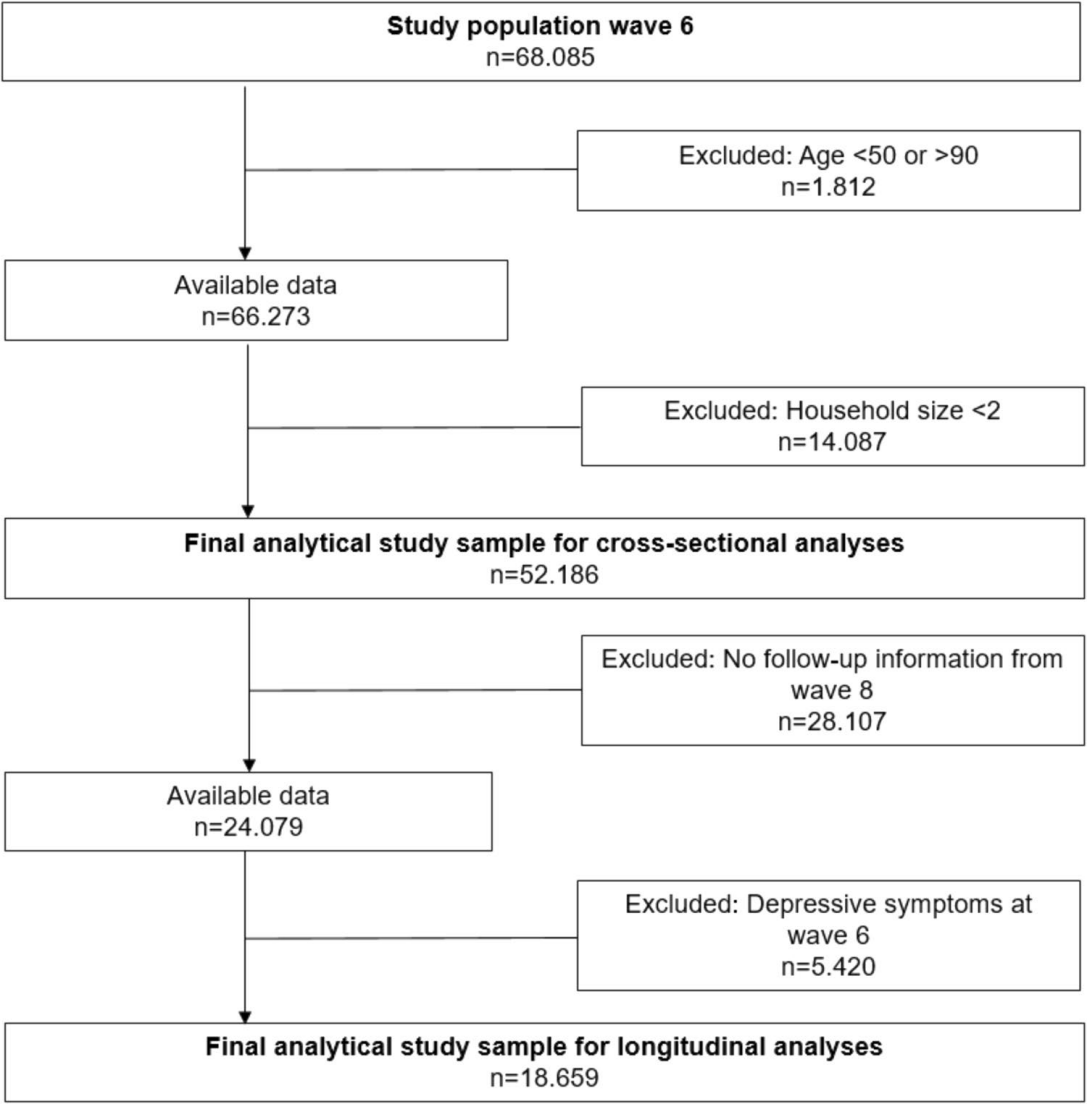
Final sample flowchart

For the cross-sectional analyses of our study, we rely on wave 6 data collected in 18 countries from 2015 to 2016. For the longitudinal analyses, wave 6 data of those who were free of elevated depressive symptoms are combined with wave 8 data to study incident depressive symptoms (available for 17 out of 18 countries). In the case of wave 8 data collection started in October 2019 and stopped in March 2020 due to the outbreak of the COVID-19 pandemic, when about 70 percent of all expected longitudinal interviews were conducted (SHARE 2022). The decision to choose these two waves is based on the fact that wave 6 is the first wave where caring was assessed together with the details of interest in our study (i.e. types and location of caring). Furthermore, the sample size of SHARE increased substantially in wave 6, because a new country joined SHARE (Croatia) and because a majority of countries that were already part of the survey had refreshment samples at this wave (9 out of 17 countries). The 18 countries included in wave 6 were: Austria, Belgium, Croatia, Czech Republic, Denmark, Estonia, France, Germany, Greece, Israel, Italy, Luxembourg, Poland, Portugal (not available in wave 8), Spain, Sweden, Switzerland, and Slovenia. Because wave 7 does not provide data on depressive symptoms, we used wave 8 to have prospective information on depressive symptoms (no data available for Portugal). For the analyses, data were restricted as follows: First, we restricted available data from wave 6 (*n* = 68.085) to people aged 50–90 years, as we were interested in caring in later life and because people older than 90 years represent a rather selective group. Second, individuals living alone were excluded, as these were not eligible to provide care inside the household. This resulted in a final analytical sample for the cross-sectional analyses of 52.186 respondents (25.122 men and 27.064 women). Of these, 18.659 respondents were free of elevated depressive symptoms in wave 6 and were included in our longitudinal analysis, thus, allowing us to apply a standard epidemiological cohort design (Rothman 2012) to investigate incident depressive symptoms and help to assure that the exposure variable precedes a potential health change (as one important criterion for causality). Details on sample selection for cross-sectional and longitudinal analyses are summarized as flowchart in Fig. 1.

### Variables

#### Caring situations

As part of the SHARE questionnaire, respondents were asked if there is someone in their household to which they provided care during the past 12 months (i.e. “in-home care”). They were also asked for the same time frame about caring outside the household (i.e. “outside-home care”). While the question on caring inside the household focusses on personal care, respondents who said that they provided care outside the household additionally specified the types of care they provided (referring to up to three persons who were cared for), ranging from personal care, practical household help to help with paperwork. This resulted in one binary indicator for personal care inside the household and another for care outside the household (irrespectively of type provided). In addition, based on information on the types of care provided outside the household, we also created three binary indicators measuring for each of the type whether it was provided or not as part of the outside-home care. This strategy both allows us to compare inside-home and outside-home personal care, as well as to compare different types of outside-home care. The exact wordings of the used questions are presented in Table S1.

#### Mental health

Poor mental health was measured in terms of increased depressive symptoms by the EURO-D depression scale. This scale includes in total 12 items for measuring the presence (based on binary indicators) of the following depressive symptomatology (referring to the past month): “depressed mood”, “pessimism”, “suicidality”, “guilt”, “sleep quality”, “interest”, “irritability”, “appetite”, “fatigue”, “concentration”, “enjoyment”, and “tearfulness”. When summing up the number of symptoms, the scale ranges from 0 to 12, with higher values indicating higher degree of depression. For the analyses, we defined more than 3 symptoms as increased levels. This cut-point has been validated against standardized psychiatric interviews in older populations (Castro-Costa et al. 2008) and has been shown to be strongly associated with other measures of depression in cross-European studies (Prince et al. 1999). As part of sensitivity analyses (presented in the supplementary material), we also considered three subscales of the EURO-D scale and explored if associations hold across sub-dimensions or were driven by one specific sub-dimension. These are two subscales (each based on three items) that were identified as part of a two-factor solution in psychometric analyses of the scale, one covering “affective suffering” and another “motivation” (Prince et al. 1999; Guerra et al. 2015), and a third subscale that includes the somatic factors of the scale (see Table S3 in the supplemental material for details).

#### Additional variables

In addition to sex and age, we included the employment situation, an indicator of respondents’ functional limitations and two socioeconomic indicators: wealth and education. Wealth is based on household total net worth, both including financial wealth (savings, net stock value, mutual funds, and bonds) and housing wealth (value of primary residence, other real estates, own business share, and cars). For the analyses, we calculated country-specific tertiles (low, medium, and high) for the cross-sectional study population. Because our wealth measure is based on accumulated savings and not on direct income, it may be more appropriate for older populations as an indicator of financial circumstances. Education is measured according to the International Standard Classification of Educational Degrees (ISCED-97) and was regrouped into "low education" (pre-primary, primary, or lower secondary education), "medium education" (secondary or post-secondary non-tertiary education), and "high education" (first and second stage of tertiary education) (UNESCO-UIS 2006). As a measure of functional limitations, we used increased number of limitations in performing instrumental activities of daily living (“IADL limitations”) based on six essential activities of an independent life. For the analyses, functional limitations were defined as having at least one IADL limitation (Lawton and Brody 1969). The employment situation measures if respondents are in paid work or not.

### Analytical strategy

All analyses are conducted for men and women separately, and we start with a sample description of the cross-sectional and the longitudinal sample (Table 1) followed by a cross table to investigate patterns of caring by sociodemographic and socioeconomic conditions (Table 2). Table 3 then gives a first answer to our main research question and presents for each caring situation (comparing carers with non-carers) the prevalence of increased depressive symptoms in the cross-sectional sample, together with the proportion of incident increased depressive symptoms in the longitudinal sample.

**Table 1:** Distribution of sociodemographic characteristics and characteristics of the caring situation in the cross-sectional (*n* = 52.186) and longitudinal (*n* = 18.659) study sample for men and women: observations (Obs.) and percentages (%)

		Cross-sectional				Longitudinal			
		Men (*n* = 25.122)		Women (*n* = 27.064)		Men (*n* = 9.550)		Women (*n* = 9.109)	
	Categories	Obs.	%	Obs.	%	Obs.	%	Obs.	%
Age	50–64 years	10.668	42.5	13.992	51.7	4.226	44.3	4.855	53.3
	65–79 years	11.710	46.6	10.923	40.4	4.684	49.1	3.900	42.8
	80–90 years	2.744	10.9	2.149	7.9	640	6.7	354	3.9
Wealth	Low	8.104	32.3	9.301	34.4	2.682	28.1	2.675	29.4
	Medium	8.378	33.3	9.020	33.3	3.205	33.6	3.051	33.5
	High	8.640	34.4	8.743	32.3	3.663	38.4	3.383	37.1
Education^a^	Low	8.825	35.7	11.119	41.7	2.663	28.3	2.964	32.9
	Medium	9.874	39.9	10.009	37.6	2.095	43.5	3.781	42.0
	High	6.029	24.4	5.516	20.7	2.647	28.1	2.256	25.1
Functional limitations^a^	Yes	3.288	13.1	4.768	17.7	599	6.3	828	9.1
	No	21.787	86.9	22.244	82.3	8.948	93.7	8.273	90.9
Employment situation^a^	In paid work	6.858	27.4	6.962	25.9	2.905	30.5	2.628	29.0
	Not in paid work	18.135	72.6	19.966	74.1	6.619	69.5	6.447	71.0
Personal care inside the household^a^	Yes	1.760	7.0	2.544	9.5	582	6.3	817	9.4
	No	23.218	93.0	24.364	90.5	8.728	93.7	7.841	90.6
Care outside the household (overall)^a^	Yes	6.809	27.2	7.394	27.4	2.759	28.9	2.520	27.7
	No	18.245	72.8	19.601	72.6	6.780	71.1	6.584	72.3
Personal care outside the household^a^	Yes	879	3.5	2.333	8.6	256	2.7	717	7.9
	No	24.175	96.5	24.662	91.4	9.293	97.3	8.390	92.1
Household help outside the household^a^	Yes	5.823	23.2	6.001	22.2	2.339	24.5	2.003	22.0
	No	19.231	76.8	20.994	77.8	7.210	75.5	7.104	78.0
Paperwork outside the household^a^	Yes	1.988	7.9	2.605	9.6	831	8.7	974	10.7
	No	23.066	92.1	24.390	90.4	8.708	91.3	8.130	89.3
Depressive symptoms (in wave 6)^b^	Not elevated	19.402	81.9	17.688	68.1				
	Elevated	4.300	18.1	8.280	31.9				

^a^For these variables, there was a proportion of missing values of below 4%

^b^2.516 participants had missing values for depressive symptoms (5.1%) in the cross-sectional study sample

**Table 2:** Proportions of carers in different caring situations by sociodemographic and socioeconomic characteristics in the cross-sectional study sample for men and women: prevalence of carers in percent (%)

		Men					Women				
	Categories	Personal care inside	Care outside (overall)	Personal care outside	Household help outside	Paperwork outside	Personal care inside	Care outside (overall)	Personal care outside	Household help outside	Paperwork outside
		%	%	%	%	%	%	%	%	%	%
Age	50–64 years	5.6	33.7	4.6	29.2	10.9	7.8	35.4	11.5	29.0	13.8
	65–79 years	7.0	24.9	2.8	21.0	6.4	10.6	21.0	6.1	16.7	5.9
	80–90 years	12.9	11.7	2.0	9.4	3.1	14.5	7.7	2.6	5.7	1.6
Wealth	Low	9.2	23.6	3.3	20.6	5.9	11.3	24.2	7.8	19.6	7.9
	Medium	6.1	27.0	3.1	23.4	7.7	9.3	26.6	8.2	21.8	9.7
	High	5.9	30.7	4.1	25.5	10.1	7.6	31.6	10.0	25.4	11.5
Education	Low	8.6	17.3	2.8	14.9	4.1	11.3	18.1	6.7	14.4	5.2
	Medium	6.5	31.0	3.4	27.4	7.9	8.7	31.7	9.4	26.1	11.0
	High	5.8	35.6	4.9	28.8	13.6	6.7	38.7	11.5	31.4	16.4
Functional limitations	Yes	14.3	14.8	2.3	11.8	4.2	15.8	17.2	6.0	13.5	5.1
	No	6.0	29.1	3.7	25.0	8.5	8.1	29.6	9.2	24.1	10.6
Employment situation	In paid work	4.7	35.4	4.6	30.2	12.2	6.1	39.3	12.3	32.3	16.8
	Not in paid work	7.9	24.1	3.1	20.6	6.3	10.6	23.3	7.4	18.7	7.2

**Table 3:** Associations between different caring situations and levels of depressive symptoms for men and women: prevalence of elevated depressive symptoms or incidence of elevated levels of depressive symptoms (cumulative incidence)

		Cross-sectional		Longitudinal	
		Men (*n* = 23.690)	Women (*n* = 25.952)	Men (*n* = 9.543)	Women (*n* = 9.098)
	Categories	Preval.	Preval.	Incid.	Incid.
Personal care inside the household	Yes	30.5	47.4	17.3	26.0
	No	17.2	30.3	12.8	19.8
Care outside the household (overall)	Yes	16.0	30.4	10.7	18.1
	No	19.0	32.5	14.2	21.2
Personal care outside the household	Yes	21.7	35.7	13.8	21.5
	No	18.0	31.5	13.0	20.1
Household help outside the household	Yes	15.5	29.8	10.4	18.1
	No	19.0	32.5	14.1	21.0
Paperwork outside the household	Yes	18.1	31.6	10.8	17.7
	No	18.1	31.9	13.3	20.5

All differences were significantly different (p-values < 0.05) based on chi^2^ tests, with except of outside-home paperwork in cross-sectional analyses and outside-home personal care in longitudinal analyse (men and women in both cases)

Next, we estimate a series of Poisson regression models to test the cross-sectional and longitudinal (Table 4) associations between caring situations and depressive symptoms. We used modified Poisson regression models with robust variance to estimate prevalence ratios (for cross-sectional analyses) and relative risks (for longitudinal analyses) (Zou 2004). Poisson regressions are an alternative to logistic regression that enable the estimation of measures of associations that are easier to interpret than odds ratios, specifically when outcomes of interest are not uncommon (Barros and Hirakara 2003). The Results section presents respective estimates together with confidence intervals (95%) and shows the average marginal effects (AMEs) based on the “margins” procedure in Stata (Williams 2012). AMEs quantify the predicted differences in proportions between categories of caring situations. For example, if the AME for in-home caring is 0.132 (with “no in-home caring” as reference group), this means that the prevalence of increased depressive symptoms is predicted to be 13.2 percentage points higher for carers compared with non-carers. All multivariable models were adjusted for age (linear and squared), education, wealth, functional limitations, employment situation, and country affiliation (broken into country dummies).

**Table 4:** Cross-sectional and longitudinal associations between different caring situations and depressive symptoms for men and women: adjusted prevalence ratios (PR) or relative risks (RR), confidence intervals (95% CI), and average marginal effects (AME)

		Cross-sectional						Longitudinal					
		Men (*n* = 23.245)			Women (*n* = 25.471)			Men (*n* = 9.368)			Women (*n* = 8.951)		
	Categories	PR	95% CI	AME	PR	95% CI	AME	RR	95% CI	AME	RR	95% CI	AME
Personal care inside the household	Yes	1.37	[1.27–1.48]	0.064	1.35	[1.29–1.41]	0.107	1.15	[0.95–1.40]	0.019	1.13	[1.02–1.30]	0.027
	No (Ref.)	–			–			–			–		
Care outside the household (overall)	Yes	1.08	[1.01–1.15]	0.014	1.12	[1.07–1.16]	0.036	0.91	[0.80–1.03]	− 0.011	1.02	[0.92–1.12]	0.002
	No (Ref.)	–			–			–			–		
Personal care outside the household	Yes	1.32	[1.16–1.51]	0.058	1.23	[1.16–1.30]	0.072	1.15	[0.88–1.50]	0.019	1.19	[1.03–1.37]	0.037
	No (Ref.)	–			–			–			–		
Household help outside the household	Yes	1.05	[0.98–1.13]	0.009	1.10	[1.05–1.15]	0.032	0.88	[0.77–1.01]	− 0.016	1.02	[0.92–1.13]	0.005
	No (Ref.)	–			–			–			–		
Paperwork outside the household	Yes	1.24	[1.13–1.37]	0.043	1.17	[1.10–1.24]	0.054	0.99	[0.80–1.21]	− 0.002	1.03	[0.89–1.20]	0.007
	No (Ref.)	–			–			–			–		

Models are calculated separately for each caring situation, adjusted for age (linear and squared), wealth, education, limitations in instrumental activities of daily living, employment situation, and country affiliation.

**Fig. 2:**
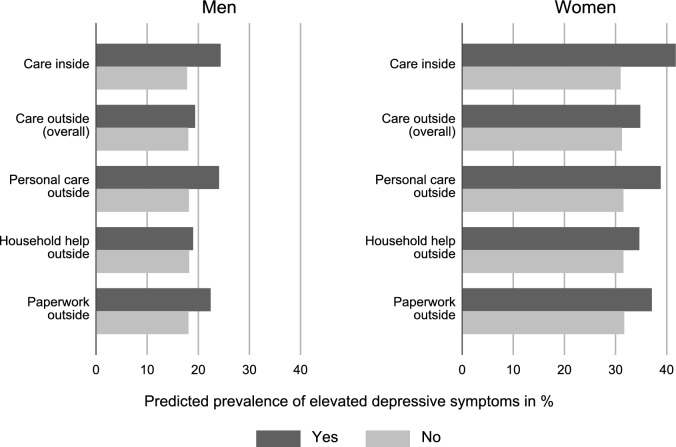
Predicted prevalence (%) of elevated levels of depressive symptoms in percent in different caring situations. Based on regression models in Table 4

**Fig. 3:**
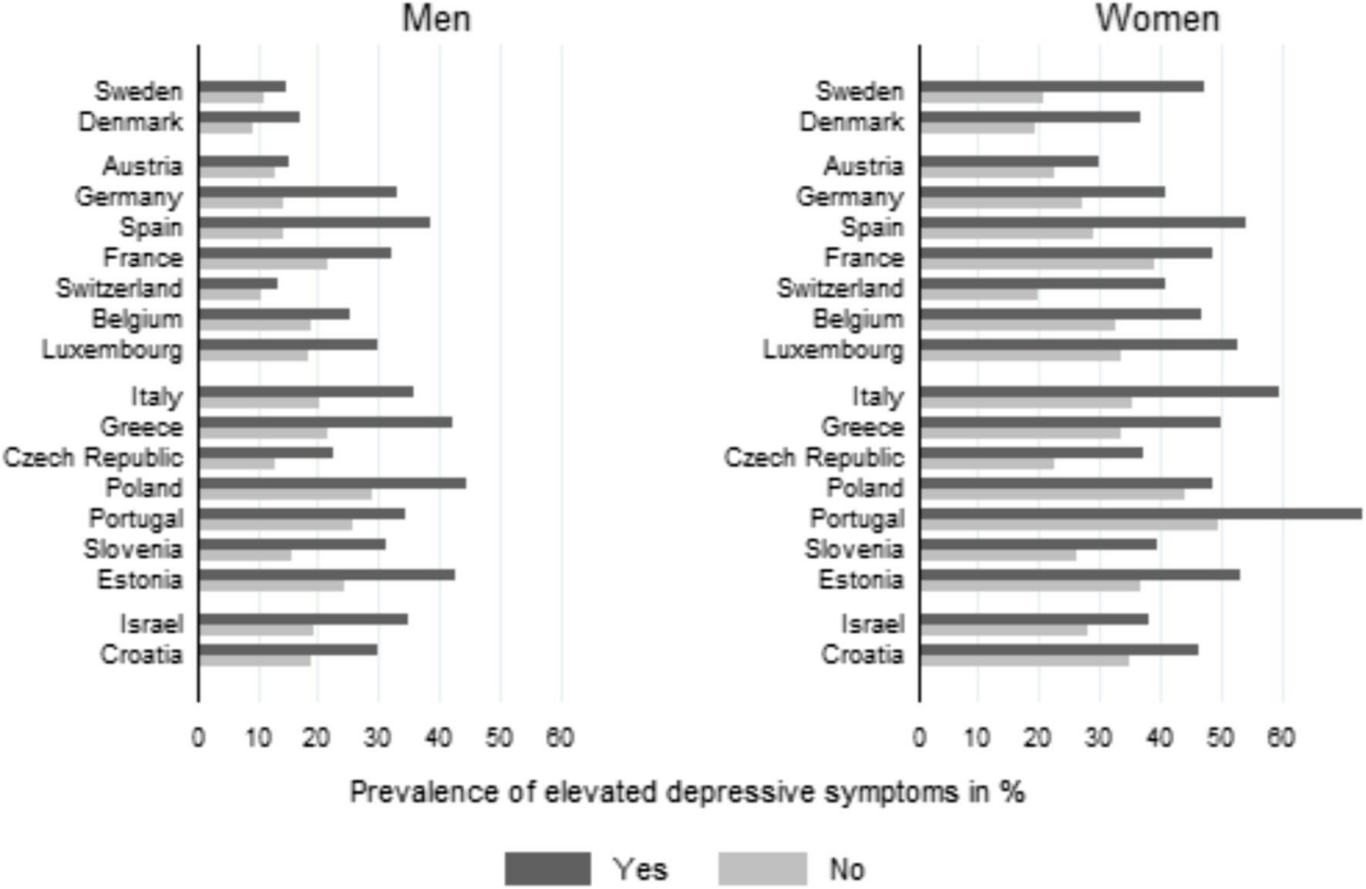
Prevalence of elevated levels of depressive symptoms in percent by personal care inside the household and country

To summarize our main findings, Fig. 2 presents the predicted prevalence of increased depressive symptoms (i.e. average adjusted predictions) by all situations of care based on the regression models. Finally, Fig. 3 presents differences in the prevalence of depressive symptoms by in-home personal care to check for constancy of findings between countries.

As part of sensitivity analyses, we recalculated longitudinal models to account for possible changes between the two waves, that is, considered if people were continuously caring/non-caring in both waves, or stopped/started caring between the two waves (see supplementary Table S2). Also, we recalculated cross-sectional analyses for the three sub-dimensions of the EURO-D scale (with same adjustments, presented in supplementary Table S3).

All calculations and figures were produced with Stata (Version 18.0).

## Results

There are slightly fewer men than women in the cross-sectional sample (25.122 men and 27.064 women). The majority are not in paid employment, and most respondents are younger than 80 (with an average age of 66). Regarding the prevalence of caring according to location, caring for someone living outside the own household (“outside-home care”) is with values of around 27% much more common than personal care inside the household (“in-home care”; below 10% for both sexes). Among the different types of outside care, household help is clearly the most common with values above 20% for both men and women. We also see that women are more likely to provide personal care, especially if it is provided outside the household. Based on our definition of elevated depressive symptoms, we see that the prevalence of such symptoms in the cross-sectional sample is clearly higher among women than men (32% vs. 18%). When comparing the cross-sectional and longitudinal sample (those who were free of elevated depressive symptoms in wave 6 and participated again in wave 8), we see that the longitudinal sample-in line with the restrictions that we applied-is a rather selective healthier sample, generally younger and less likely to report functional limitations.

Table 2 shows that there are sociodemographic and socioeconomic differences in caring, with results clearly varying according to the location where caring takes place. In particular, while older respondents are more likely to provide personal in-home care, they are less likely to provide all types of outside care (both men and women). In addition, people with lower levels of education or wealth provide less outside care, but are more likely to provide in-home care. Likewise, in-home care is more common in the case of functional limitations, while the opposite holds for outside care. These differences highlight the importance of adjusting for these factors in multivariable analyses.

A first answer to our main research questions is presented in Table 3 in the form of bivariate associations between care situations and elevated depressive symptoms. Both men and women providing personal care inside the household are more likely to report elevated depressive symptoms (cross-sectional findings) and are more likely to develop elevated depressive symptoms between the two waves (longitudinal findings). For example, when looking at the longitudinal sample, we see that 26% of women and 17.3% of men who provided in-home personal care at wave 6 (and were free of elevated symptoms at that time) had elevated depressive symptoms in wave 8, while values were 19.8% and 12.8% for respective non-carers. This corresponds to a difference in percentage points of 6.2 points for women and of 4.5 points for men. In the case of outside care, we observe that those providing care (especially household help) have better mental health than those providing in-home personal care. Yet, personal care is associated with poorer mental health also when provided outside the household.

Results testing these associations (and adjusting for covariates) are presented in Table 4. Overall, the findings for personal inside care remain constant even after adjusting for age, education, wealth, functional limitations, employment situation, and country affiliation. Specifically, for the cross-sectional findings, the AMEs indicate that the prevalence of elevated depressive symptoms is 6.4 percentage points higher for male carers compared with males who do not provide personal inside care. The respective difference for female carers is 10.7 percentage (with strong evidence against the null hypotheses). In the case of the longitudinal analyses, the respective estimates again suggest that men and women providing personal inside care are at increased risk of developing elevated depressive symptoms, with relative risks of 1.15 and 1.13, respectively. For outside care, it is worth noting that we now observe no association for household help, suggesting that the positive association in the bivariate analyses was largely driven by better socioeconomic circumstances and better health. For personal care and paperwork, we observe cross-sectional but not longitudinal associations with poorer mental health for carers compared to non-carers of outside care. The AMEs indicate that the prevalence of elevated depressive symptoms in personal care outside the household is 5.8 percentage points higher for male carers and 7.2 higher for female carers compared with non-caring counterparts. Comparing carers and non-carers for help with paperwork outside the household, the prevalence of elevated depressive symptoms is 4.3 percentage points higher for male and 5.4 higher for female carers. However, overall, strongest cross-sectional associations between caring and mental health are found for respondents who provide personal care inside their own household.

When estimating the association for each sub-dimension of the EURO-D scale separately, results were similar, with slightly stronger results in the case of “affective functioning” which includes psychological symptoms in the form of depressive mood and negative feelings (Table S3). Furthermore, sensitivity analyses that considered changes in caring state between the two waves (see supplementary Table S2) support our results. These analyses indicated an elevated risk of depressive symptoms for people who provided care for someone inside the own household at both waves or who started providing inside care between the two waves-but not for those who stopped providing inside care between the two waves (again for both men and women).

Fig. 2 summarizes the main cross-sectional findings across all countries (based on the regression models in Table 4) and again shows that elevated levels of depressive symptoms are particularly high for men and women providing care inside the household and for those providing personal care outside the household. In addition, as shown in Fig. 3, we see that our main findings of a negative association between in-home care and poor mental health are consistent throughout countries (with overall varying levels of poor mental health by sex and countries).

## Discussion

This study provides new insights into the complex relationships between caring and mental health, by showing that location and type of care are important characteristics of the care situation that affect its association with mental health. Specifically, we found that for both men and women, personal care provided inside the household was associated with elevated depressive symptoms. This association was found in both cross-sectional and longitudinal analyses, thus, providing additional support that the associations are not simply due to selection into caring. Furthermore, our research underscores the need to differentiate between various forms of caring. We found that personal care provided outside the household, though to a lesser degree than personal care inside the household, is more likely to be associated with negative mental health outcomes than household help. Likewise, assisting with paperwork was associated with more elevated depressive symptoms than household help-a finding that probably points to cognitive decline and serious health issues of the care recipient.

Another important descriptive finding of our study includes the varying socioeconomic gradients in caring. Men and women with lower education and wealth were more likely to provide care inside the household, yet less likely to provide any type of care outside the household. Similarly, people who provided care inside the household tended to be older and have functional limitations, whereas those providing outside care were generally younger and had fewer functional limitations.

Overall, our findings are in line with the previous studies, especially studies that have documented poorer mental health among people providing personal care (without considering the location) (Hansen and Slagsvold 2013). This finding may stem from the challenging psychosocial conditions of personal care, often marked by limited rewarding exchanges and diminished feelings of self-efficacy (Verbakel et al. 2017; Hiel et al. 2015; McMunn et al. 2009; Wahrendorf et al. 2008). Additionally, the necessity (not the choice) of providing personal care due to a close relative's health could be a significant factor. Besides psychobiological stress, the inherent burden and worries of having a relative in poor health is also a crucial aspect associated with the carers' mental well-being (Litwin and Stoeckel 2014; Hansen et al. 2013). This is also supported by our finding that we found no association for household help, which is probably also due to the overall better health conditions of the care recipients, rather than of the caring activity per se. Or, as we have seen in our descriptive analyses, personal care is often accompanied by socioeconomic disadvantages that are also negatively related to health. Another finding of our study was that care provided outside the household seemed to be generally less consistently associated with poor mental health than care inside the household. Here, we can speculate that the more pronounced association for in-home care is related to an additional burden of care, due to limited opportunities to recover or to distance oneself from caring duties. In a similar way, this idea is currently discussed as "blurring boundaries" in the context of work stress research (Simenenko and Lentjushenkova 2022; Eurofound and the International Labour Office 2017).

Our study adds to current cross-sectional and longitudinal knowledge in at least three ways: First, by using a comprehensive assessment of the care situation, taking into account both the location and type of caring, we were able to make a systematic comparison of the impact of different care situations, whereas the previous studies have often lumped together different types of care or focussed on only one aspect of the care situation. This distinction indicates that the associations between outside care and carer mental health are clearly depending on the type of care provided. It also suggests that previous findings of a positive association for outside care and mental health (Kaschowitz and Brandt 2017) are likely to reflect that outside care is mostly provided in the form of household help. Second, our sex-specific analysis revealed similar associations between caring and mental health for both men and women. This finding is notable as several, mostly qualitative, studies have reported differing caring experiences for men and women (Swinkels et al. 2019; Gallicchio et al. 2002; Zygouri et al. 2021). However, most quantitative studies, possibly due to the smaller sample sizes of male carers, have not made such distinctions. This points to a gap in quantitative research on sex-specific caring experiences. Third, by showing that more advantaged population groups and those without functional limitations are likely to provide care outside the household, our findings support the idea that positive health associations in other studies may partly stem from these carers’ better socioeconomic and health situation. Conversely, disadvantaged groups tend to offer in-home care, which is linked to poorer health. This suggests a compounding of disadvantages, where societal and health inequalities are further exacerbated by the demands of caring, aligning with the theory of risk accumulation (Dannefer 2003).

The following limitations need to be considered when interpreting the results. First, our longitudinal analyses used caring data from SHARE wave 6 (referring to the previous 12 months) and depressive symptoms 4 years later. In doing so, we could not distinguish between short or long periods of caring, as well as it remains unclear if and for how long respondents remained carers beyond wave 6. These aspects might affect the reliability of our measure of caring, specifically in the case of our longitudinal analyses. Although additional sensitivity analyses (see supplementary Table S2) support our results, the absence of detailed changes in caring (or on “caring histories”) between the waves is a notable limitation of the longitudinal analyses. Likewise, while the provision of care referred to the past year, depressive symptoms were assessed for the past month. Although some may argue that this helps to assure that our exposure precedes the outcome in the cross-sectional analyses (and partly helps to address reverse causality), it may also introduce bias. Specifically, as personal care (to a seriously ill person) is often provided for a short-term period only, many carers may no longer provide care when reporting depressive symptoms, and therefore report better mental health than while providing care (because of being relieved of caring). As a result, we may have underestimated the impact in our study. Likewise, when comparing cross-sectional and longitudinal, we clearly see that the longitudinal sample is comparatively healthier in terms of functional limitations. This is surely also due to our selection strategy enabling us to study incident depressive symptoms based on the longitudinal sample, and thus, to apply an epidemiological study design that addresses analytical (i.e. causal) questions (Stöckel and Bom 2022; Kolodziej et al. 2022). But it may also be the case that the healthier sample is due to selective attrition where people with poor mental health are less likely to participate at wave 8. This again could mean that we have underestimated the impact of caring (specifically in-home personal care) on mental health. At this point, it should also be noted that we focussed on an increase of depressive symptoms between the two measurement points, but that our strategy did not allow to study potential improvement of mental health. Next, our study faces limitations due to varying response rates and potential sample selectivity in SHARE, particularly among younger individuals and those in paid work. This might lead to an underestimation of caring prevalence. While SHARE provides weights to address non-response and attrition, we choose not to use them as our focus was not on quantifying care prevalence and due to missing weight values. Additionally, use of weights in multivariable models are debated (Winship and Radbill 1994), especially when models include similar variables that are used to create the weights (e.g. country, sex, and age). Nevertheless, our separate analyses with weights, accounting for complex survey designs, yielded nearly identical results. Third, although we adjusted for country affiliation (thus accounting for varying levels of depressive symptoms by country), we could still ask whether the associations between caring and mental health were similar for each country. In particular, national policies related to long-term care (LTC policies), such as national spending on long-term care or availability of paid leave (but also the degree of “defamilialisation”), could modify the extent to which care provision and health are related (Saraceno and Keck 2010; Verbakel 2014; Verbakel et al. 2023). For example, some studies suggest that associations are less pronounced in the case of extended supportive policies (Verbakel 2014; Uccheddu et al. 2019; Brenna and Di Nova 2016). However, this study aimed to add to the literature by explicitly considering location and type of care when investigating sex-specific associations with mental health. In this context, an in-depth analysis of different national policies would probably require a focus on one specific care situation (e.g. in-home personal care) in relation to health (with sex-specific analyses and with longitudinal analyses). Nevertheless, we find some support that our main results of an negative association between in-home personal care and depressive symptoms are similar for men and women within all countries under study, including countries marked by defamilialisation (Denmark and Sweden) and countries where the family has traditionally played an important role in the provision of care (e.g. Italy and Greece, located in the lower half of Fig. 3). Finally, in this study, we have taken into account the location and type of care. There are, of course, other aspects of the care situation that may be of interest, such as the intensity of care, the relationship of the carer to the person being cared for, details of the person's health conditions, or even information on whether the carer received professional support or support from other persons. Especially regarding the intensity of care, many studies have shown that a high intensity is associated with poorer mental health outcomes (e.g. Kolodziej et al. 2022; Bom and Stöckel 2021). However, this information is either not available in SHARE (or only for selective waves) or would rather require an additional study with a different focus.

In conclusion, our findings highlight that both the location and the nature of caring are crucial for grasping the intricate link between caring and the mental health of carers. Findings indicate a detrimental impact of especially personal care for both male and female carers across Europe, particularly when provided within the carer’s own household. Furthermore, our findings suggest that older and socially disadvantaged populations are more likely to provide care under such circumstances, and that associations remain consistent after adjusting for socioeconomic conditions. These findings call for increased intervention efforts among more disadvantaged population groups providing care inside the household, for example, by improving the care situation through formal care assistance, offering respite options, professional care training, or psychological counselling.

## Supplementary Information

Below is the link to the electronic supplementary material.

Supplementary file1 (DOCX 46 KB)

## Data Availability

This paper uses data from SHARE Waves 1, 2, 3, 4, 5, 6, 7, and 8 (DOIs: 10.6103/SHARE.w1.900, 10.6103/SHARE.w2.900, 10.6103/SHARE.w3.900, 10.6103/SHARE.w4.900, 10.6103/SHARE.w5.900, 10.6103/SHARE.w6.900, 10.6103/SHARE.w6.DBS.100, 10.6103/SHARE.w7.900, 10.6103/SHARE.w8.900) see Börsch-Supan et al. (2013) for methodological details. The SHARE data collection has been funded by the European Commission, DG RTD through FP5 (QLK6-CT-2001-00360), FP6 (SHARE-I3: RII-CT-2006-062193, COMPARE: CIT5-CT-2005-028857, SHARELIFE: CIT4-CT-2006-028812), FP7 (SHARE-PREP: GA N°211909, SHARE-LEAP: GA N°227822, SHARE M4: GA N°261982, DASISH: GA N°283646), and Horizon 2020 (SHARE-DEV3: GA N°676536, SHARE-COHESION: GA N°870628, SERISS: GA N°654221, SSHOC: GA N°823782, SHARE-COVID-19: GA N°101015924) and by DG Employment, Social Affairs & Inclusion through VS 2015/0195, VS 2016/0135, VS 2018/0285, VS 2019/0332, VS 2020/0313, SHARE-EUCOV: GA N°101052589 and EUCOVII: GA N°101102412. Additional funding from the German Federal Ministry of Education and Research (01UW1301, 01UW1801, 01UW2202), the Max Planck Society for the Advancement of Science, the U.S. National Institute on Aging (U01_AG09740-13S2, P01_AG005842, P01_AG08291, P30_AG12815, R21_AG025169, Y1-AG-4553-01, IAG_BSR06-11, OGHA_04-064, BSR12-04, R01_AG052527-02, R01_AG056329-02, R01_AG063944, HHSN271201300071C, RAG052527A) and from various national funding sources is gratefully acknowledged (see www.share-eric.eu).
