# Supplementary material for: How are location and type of caring associated with the carer’s mental health? Cross-sectional and longitudinal findings from SHARE
Source: Eur J Ageing. 2025 Feb 21;22(1):5. doi: 10.1007/s10433-025-00843-3 (PMC11845335; doi:10.1007/s10433-025-00843-3)
Supplement: Supplementary file 1 — Supplementary file1 (DOCX 46 KB) [file 10433_2025_843_MOESM1_ESM.docx]

**Supplementary Material**

Table S1: Questions in SHARE related to caring

| **Variables** | **Question** | **Answer options** |
| --- | --- | --- |
| Care **inside** the household (personal care only) | Is there someone **living in this household** whom you have helped regularly during the last twelve months with **personal care**, such as washing, getting out of bed or dressing? | Yes  No |
| Care **outside** the household  (overall) | In the last twelve months, have you personally given any kind of help (personal care, practical household help or help with paperwork) to a family member from **outside** the household, a friend or neighbor? | Yes  No |
| Type of care **outside** the household | Which types of help have you given to this person in the last twelve months? | 1. **Personal care**, e. g. dressing, bathing or showering, eating, getting in or out of bed, using the toilet  2. **Practical household help**, e. g. home repairs, gardening, transportation, shopping, household chores  3. **Help with paperwork**, e. g. filling out forms, setting financial or legal matters |

Table S2: Adjusted longitudinal associations between trajectories of caring situations and depressive symptoms for men and women:
Incident cases of elevated levels of depressive symptoms (Cases), cumulative incidence in %, relative risks (RR),
confidence intervals (95% CI) and average marginal effects (AME)

|  |  | Men | | | | | Women | | | | |
| --- | --- | --- | --- | --- | --- | --- | --- | --- | --- | --- | --- |
|  | Categories | Cases | % | RR | 95% CI | AME | Cases | % | RR | 95% CI | AME |
|  |  |  |  |  |  |  |  |  |  |  |  |
| Personal care inside the household | Caring at both waves | 25 | 21.55 | 1.43 | [1.00-2.04] | 0.051 | 64 | 27.83 | 1.27 | [1.02-1.58] | 0.051 |
|  | Giving up (only wave 6) | 60 | 16.04 | 1.14 | [0.89-1.46] | 0.018 | 83 | 24.56 | 1.16 | [0.95-1.41] | 0.030 |
|  | Starting (only wave 8) | 92 | 19.83 | 1.42 | [1.17-1.72] | 0.052 | 194 | 33.11 | 1.59 | [1.40-1.81] | 0.112 |
|  | No caring at both waves (Ref.) | 1.014 | 12.18 | - | - | - | 1.388 | 18.58 | - | - | - |
|  |  |  |  |  |  |  |  |  |  |  |  |
| Care outside the household (overall) | Caring at both waves | 132 | 9.13 | 0.84 | [0.70-1.02] | -0.022 | 221 | 17.53 | 1.07 | [0.93-1.23] | 0.013 |
|  | Giving up (only wave 6) | 196 | 12.10 | 0.92 | [0.79-1.06] | -0.012 | 278 | 18.53 | 1.02 | [0.90-1.15] | 0.004 |
|  | Starting (only wave 8) | 138 | 10.54 | 0.87 | [0.73-1.04] | -0.018 | 247 | 19.65 | 1.08 | [0.95-1.23] | 0.017 |
|  | No caring at both waves (Ref.) | 777 | 15.07 | - | - | - | 1.094 | 21.56 | - | - | - |
|  |  |  |  |  |  |  |  |  |  |  |  |
| Personal care outside the household | Caring at both waves | 5 | 15.62 | 1.31 | [0.58-2.95] | 0.040 | 39 | 24.38 | 1.35 | [1.02-1.79] | 0.069 |
|  | Giving up (only wave 6) | 42 | 13.59 | 1.14 | [0.86-1.50] | 0.018 | 123 | 20.71 | 1.16 | [0.98-1.36] | 0.031 |
|  | Starting (only wave 8) | 27 | 12.05 | 1.07 | [0.72-1.53] | 0.009 | 112 | 20.14 | 1.11 | [0.93-1.32] | 0.022 |
|  | No caring at both waves (Ref.) | 1.171 | 13.04 | - | - | - | 1.566 | 20.11 | - | - | - |
|  |  |  |  |  |  |  |  |  |  |  |  |
| Household help outside the household | Caring at both waves | 103 | 8.84 | 0.80 | [0.65-0.99] | -0.027 | 161 | 17.60 | 1.08 | [0.83-1.41] | 0.016 |
|  | Giving up (only wave 6) | 174 | 11.58 | 0.89 | [0.76-1.04] | -0.015 | 257 | 18.41 | 1.02 | [0.90-1.15] | 0.003 |
|  | Starting (only wave 8) | 121 | 10.34 | 0.85 | [0.70-1.02] | -0.021 | 210 | 19.32 | 1.08 | [0.94-1.24] | 0.016 |
|  | No caring at both waves (Ref.) | 847 | 14.85 | - | - | - | 1.212 | 21.27 | - | - | - |
|  |  |  |  |  |  |  |  |  |  |  |  |
| Paperwork outside the household | Caring at both waves | 22 | 9.73 | 1.00 | [0.67-1.49] | 0.000 | 63 | 21.58 | 1.33 | [1.06-1.67] | 0.066 |
|  | Giving up (only wave 6) | 71 | 11.18 | 0.99 | [0.78-1.24] | -0.002 | 104 | 15.98 | 0.94 | [0.78-1.13] | -0.012 |
|  | Starting (only wave 8) | 64 | 10.58 | 1.05 | [0.82-1.33] | 0.007 | 132 | 19.35 | 1.13 | [0.96-1.33] | 0.026 |
|  | No caring at both waves (Ref.) | 1.086 | 13.46 | - | - | - | 1.541 | 20.63 | - | - | - |
|  |  |  |  |  |  |  |  |  |  |  |  |

Note: Models are calculated separately for each caring situation, adjusted for age (linear and squared), wealth, education,
limitations in instrumental activities of daily living, employment situation, and country affiliation

Table S3: Cross- and longitudinal associations between characteristics of the caring situation and three factors (affective suffering, motivation, somatic factors) of the EURO-D scale for men and women: Adjusted prevalence ratios (PR) and relative risks (RR), confidence intervals (95% CI) and average marginal effects (AME)

|  |  | Men | | | Women | | |
| --- | --- | --- | --- | --- | --- | --- | --- |
|  |  | PR | 95% CI | AME | PR | 95% CI | AME |
|  |  |  |  |  |  |  |  |
| **Affective suffering** |  |  |  |  |  |  |  |
| Personal care inside the household | Yes | 1.62 | [1.44-1.84] | 0.051 | 1.37 | [1.39-1.45] | 0.097 |
|  | No (Ref.) | - | - | - | - | - | - |
|  |  |  |  |  |  |  |  |
| Care outside the household (overall) | Yes | 1.19 | [1.08-1.31] | 0.016 | 1.18 | [1.13-1.24] | 0.047 |
|  | No (Ref.) | - | - | - | - | - | - |
|  |  |  |  |  |  |  |  |
| Personal care outside the household | Yes | 1.66 | [1.40-1.98] | 0.056 | 1.35 | [1.27-1.43] | 0.092 |
|  | No (Ref.) | - | - | - | - | - | - |
|  |  |  |  |  |  |  |  |
| Household help outside the household | Yes | 1.19 | [1.08-1.32] | 0.016 | 1.16 | [1.11-1.22] | 0.043 |
|  | No (Ref.) | - | - | - | - | - | - |
|  |  |  |  |  |  |  |  |
| Paperwork outside the household | Yes | 1.31 | [1.14-1.51] | 0.026 | 1.22 | [1.14-1.30] | 0.057 |
|  | No (Ref.) | - | - | - | - | - | - |
|  |  |  |  |  |  |  |  |
| **Motivation** |  |  |  |  |  |  |  |
| Personal care inside the household | Yes | 1.28 | [1.10-1.48] | 0.018 | 1.31 | [1.17-1.46] | 0.023 |
|  | No (Ref.) | - | - | - | - | - | - |
|  |  |  |  |  |  |  |  |
| Care outside the household (overall) | Yes | 0.77 | [0.67-0.88] | -0.016 | 0.81 | [0.72-0.91] | -0.015 |
|  | No (Ref.) | - | - | - | - | - | - |
|  |  |  |  |  |  |  |  |
| Personal care outside the household | Yes | 1.14 | [0.88-1.48] | 0.009 | 0.97 | [0.83-1.14] | -0.002 |
|  | No (Ref.) | - | - | - | - | - | - |
|  |  |  |  |  |  |  |  |
| Household help outside the household | Yes | 0.73 | [0.62-0.86] | -0.018 | 0.84 | [0.74-0.96] | -0.013 |
|  | No (Ref.) | - | - | - | - | - | - |
|  |  |  |  |  |  |  |  |
| Paperwork outside the household | Yes | 0.84 | [0.67-1.05] | -0.010 | 0.88 | [0.73-1.06] | -0.009 |
|  | No (Ref.) | - | - | - | - | - | - |
|  |  |  |  |  |  |  |  |
| **Somatic** |  |  |  |  |  |  |  |
| Personal care inside the household | Yes | 1.28 | [1.17-1.41] | 0.041 | 1.27 | [1.20-1.35] | 0.067 |
|  | No (Ref.) | - | - | - | - | - | - |
|  |  |  |  |  |  |  |  |
| Care outside the household (overall) | Yes | 1.05 | [0.97-1.13] | 0.007 | 1.08 | [1.03-1.14] | 0.021 |
|  | No (Ref.) | - | - | - | - | - | - |
|  |  |  |  |  |  |  |  |
| Personal care outside the household | Yes | 1.19 | [1.02-1.39] | 0.028 | 1.15 | [1.07-1.23] | 0.037 |
|  | No (Ref.) | - | - | - | - | - | - |
|  |  |  |  |  |  |  |  |
| Household help outside the household | Yes | 1.00 | [0.92-1.08] | -0.005 | 1.05 | [1.00-1.08] | 0.012 |
|  | No (Ref.) | - | - | - | - | - | - |
|  |  |  |  |  |  |  |  |
| Paperwork outside the household | Yes | 1.19 | [1.06-1.33] | 0.028 | 1.15 | [1.07-1.23] | 0.036 |
|  | No (Ref.) | - | - | - | - | - | - |
|  |  |  |  |  |  |  |  |

Note: Models are calculated separately for each caring situation, adjusted for age (linear and squared), wealth, education,
limitations in instrumental activities of daily living, employment situation, and country affiliation.

Affective suffering includes depressive mood, wishing death and tearfulness, motivation includes loss of interest, poor concentration and lack of enjoyment, somatic factors include sleeplessness, loss of appetite and fatigue. “Caseness“ was assumed when two or more (out of three) items applied.
